# Genetic composition of interspecific potato somatic hybrids and autofused 4*x* plants evaluated by DArT and cytoplasmic DNA markers

**DOI:** 10.1007/s00299-016-1966-2

**Published:** 2016-03-18

**Authors:** Paulina Smyda-Dajmund, Jadwiga Śliwka, Iwona Wasilewicz-Flis, Henryka Jakuczun, Ewa Zimnoch-Guzowska

**Affiliations:** Plant Breeding and Acclimatization Institute - National Research Institute, Młochów Research Center, Platanowa 19, 05-831 Młochów, Poland

**Keywords:** Diversity array technology, Nuclear genome, Protoplast fusion, *Solanum* × *michoacanum*; *Solanum tuberosum*

## Abstract

*****Key message***:**

**Using DArT analysis, we demonstrated that all*Solanum*** **×** ***michoacanum*(+)*S.tuberosum*somatic hybrids contained all parental chromosomes. However, from 13.9 to 29.6** **% of the markers from both parents were lost in the hybrids.**

**Abstract:**

Somatic hybrids are an interesting material for research of nucleus-cytoplasm interaction and sources of new nuclear and cytoplasmic combinations. Analyses of genomes of somatic hybrids are essential for studies on genome compatibility between species, its evolution and are important for their efficient exploitation. Diversity array technology (DArT) permits analysis of the composition of nuclear DNA of somatic hybrids. The nuclear genome compositions of 97 *Solanum* **×** *michoacanum* (+) *S. tuberosum* [*mch* (+) *tbr*] somatic hybrids from five fusion combinations and 11 autofused 4*xmch* were analyzed for the first time based on DArT markers. Out of 5358 DArT markers generated in a single assay, greater than 2000 markers were polymorphic between parents, of which more than 1500 have a known chromosomal location on potato genetic or physical map. DArT markers were distributed along the entire length of 12 chromosomes. We noticed elimination of markers of wild and *tbr* fusion components. The nuclear genome of individual somatic hybrids was diversified. *Mch* is a source of resistance to *Phytophthora infestans*. From 97 *mch* (+) *tbr* somatic hybrids, two hybrids and all 11 autofused 4*x mch* were resistant to *P. infestans*. The analysis of the structure of particular hybrids’ chromosomes indicated the presence of markers from both parental genomes as well as missing markers spread along the full length of the chromosome. Markers specific to chloroplast DNA and mitochondrial DNA were used for analysis of changes within the organellar genomes of somatic hybrids. Random and non-random segregations of organellar DNA were noted.

**Electronic supplementary material:**

The online version of this article (doi:10.1007/s00299-016-1966-2) contains supplementary material, which is available to authorized users.

## Introduction

A significant number of somatic hybrids of various plant species have been obtained to produce novel, intergeneric and intrageneric hybrids with new nuclear and cytoplasmic compositions, and to transfer important genes into breeding gene pools (Orczyk et al. 2003; Tiwari et al. 2010). The somatic hybridization process leads to new combinations of chloroplast and mitochondrial genomes, and generates complex interactions between genomes and plasmons (Orczyk et al. 2003; Iovene et al. 2007). Chromosomal deletions, aberrations, eliminations or recombination between homologous fragments of chromosomes are often observed after somatic fusion (Harding and Millam 2000; Orczyk et al. 2003). Information regarding diversity and the composition of a somatic hybrid genome is useful for its efficient exploitation. Detailed analysis of somatic hybrid genomes and their relation to phenotypic data can provide important information on the genetic nature of traits of interest.

The transfer of genes for resistance to biotic and abiotic stresses into the cultivated potato genome is a frequent subject of potato research. *Phytophthora infestans* (Mont.) de Bary is one of the most important potato pathogens, and resistance against this pathogen is one of the main aims of potato breeding (Park et al. 2009). Wild potato species are sources of resistance to *P. infestans*, and introgression of novel resistance genes from wild *Solanum* species into the tetraploid potato gene pool is a method to achieve progress in breeding the potato cultivars resistant to late blight (Zimnoch-Guzowska et al. 2003; Zoteyeva et al. 2012). In specific cases, transfer of desirable resistance genes is possible only through methods different than sexual hybridization due to crossing barriers. Somatic hybrids resistant to *P. infestans* were previously obtained between *Solanum tuberosum* (*tbr)* and several wild potato species (Orczyk et al. 2003; Smyda et al. 2013; Chandel et al. 2015). Of this group, only few hybrids between *tbr* and *Solanum bulbocastanum* (Helgeson et al. 1998), *Solanum nigrum* (Horsman et al. 2001), *Solanum tarnii* (Thieme et al. 2008), *Solanum commersonii* (Carputo et al. 2000), and *Solanum cardiophyllum* (Thieme et al. 2010) were subsequently backcrossed sexually to potato cultivars and exploited in potato breeding programs. Low number of hybrids suitable for an application in the breeding process have been caused by the low frequency of somatic hybrids maintaining resistance of the donor component, their reduced fertility, crossing incapability or poor tuber performance (Orczyk et al. 2003; Szczerbakowa et al. 2010).

Limited information is available on the detailed genomic composition of the potato somatic hybrids. Hybrid genome composition has been characterized using cytological or molecular techniques. Metaphase chromosomes of potato are very small, ranging in length from 1.0 to 3.5 μm (Dong et al. 2000), the chromosomes are similar to each other and lack morphological markers (Gavrilenko 2007). Thus, the application of traditional cytogenetic analyses of metaphase chromosomes in potato, which relies on chromosome differentiation using techniques such as genome in situ hybridization (GISH), fluorescence in situ hybridization (FISH) or GISH combined with FISH is quite difficult (Srebniak et al. 2002; Iovene et al. 2007). Based on these techniques, it has been possible to assess the genome composition of *Solanum villosum* (+) *tbr* (Tarwacka et al. 2013) and to detect the loss of chromosomes of *Solanum brevidens* (+) *tbr* (Gavrilenko et al. 2002) or alterations in the chromosomal structure of *S. bulbocastanum* (+) *tbr* (Iovene et al. 2007).

Alternative methods to characterize nuclear genome composition include polymerase chain reaction (PCR) or hybridization-based markers from genetic and physical maps of potato. Based on restriction fragment length polymorphisms (RFLPs) (Menke et al. 1996; Yamada et al. 1998), random amplified polymorphic DNA (RAPD) (Polgar et al. 1999; Bołtowicz et al. 2005) and simple sequence repeat (SSR) markers (Harding and Millam 2000; Chen et al. 2013) alterations in the genome content in potato somatic hybrids, chromosome elimination and recombination events between homologous chromosomes have been described. Another marker system, i.e., diversity array technology (DArT), appears to be a promising method to obtain more precise information regarding the genome composition of somatic hybrids. The process of DArT marker discovery consists of several steps: creating genomic representation, library creation, microarraying DNA fragments onto glass slides, hybridization of labeled probes, scanning and data analysis (Jaccoud et al. 2001). A critical step of DArT technology is genomic complexity reduction (Jaccoud et al. 2001). A reduced fraction of the genome is prepared by restriction enzyme digestion of genomic DNA followed by the ligation of restriction fragments to adapters. The combination of *Pst*I/*Taq*I enzymes was selected for potato marker finding (Śliwka et al. 2012a). The genome complexity is then reduced by PCR amplification. Amplicons from representations are cloned, amplified, purified and arrayed onto a glass slides. Simultaneously, the DNA probes assigned for hybridization with microarray are also digested by restriction enzymes, ligated, amplified, and then all successful amplifications products are labeled with either Cy3 or Cy5 fluorescent dye and hybridized to the microarray. Microarrays are scanned and data (0/1) are analyzed (Jaccoud et al. 2001).

DArT maps have been constructed for a variety of plant species, including rice (Jaccoud et al. 2001), thale cress (Wittenberg et al. 2005), wheat (Crossa et al. 2007), barley (Li et al. 2008), rye (Bolibok-Brągoszewska et al. 2009) and others (Varshney et al. 2010). DArT linkage maps of potato were created for *Solanum* **×** *michoacanum* (*mch*) (Śliwka et al. 2012a), *Solanumruiz*-*ceballosii* (Śliwka et al. 2012b) and a doubled haploid DM1–3 of *Solanum phureja*, for which a physical map is available, too (Sharma et al. 2013). Three additional DArT maps of diploid potato (Sołtys-Kalina et al. 2015; Śliwka et al. 2016; data unpublished, personal communication with Agnieszka Hara-Skrzypiec) are available. Using the DArT method, it is possible to generate several hundred to several thousand markers in a single assay (Kilian et al. 2005; Wittenberg et al. 2005). For example, 846 DArT markers for *mch* and 1827 DArT markers for *S. phureja* have been mapped on genetic maps of potato (Śliwka et al. 2012a; Sharma et al. 2013).

Specific markers are needed to track changes in organellar genomes. The characterization of mitochondrial DNA (mtDNA) and chloroplast DNA (cpDNA) has been reported in several *Solanum* hybrids. The cytoplasmic composition of potato somatic hybrids has been investigated through southern blot analysis with total organellar DNA and specific labeled probes for cpDNA and mtDNA restriction-profile analysis (Bastia et al. 2001). The PCR–based molecular markers specific to cytoplasmic DNA were developed by Lössl et al. (1999, 2000) and Hosaka and Sanetomo (2012). Hosaka and Sanetomo (2012), grouped potato cytoplasm into six types: T (*S. tuberosum* ssp. *tuberosum*), D (*Solanumdemissum*), P (*S.phureja*), A (*S. tuberosum* ssp. *andigena*), M (Mother type) and W (Wild species). This categorization was based on the combinations of five markers: T, S, SAC, D and A. Markers T, S, SAC and A are specific to cpDNA, whereas the D marker is specific to *S. demissum* and indicates its mitochondrial origin (Sanetomo and Hosaka 2013). M type cytoplasm based on Hosaka and Sanetomo (2012) is C/ε according to an older system created by Lössl et al. (2000). P, A, T, D are S/ε, A/ε, T/ß and W/α, respectively. W type based on additional ALM_4 and ALM_5 markers can be divided into three types: W/α, W/ß and W/γ. The chloroplast genomes of somatic hybrids have been inherited from one of the protoplast parental forms in previously examined *Solanum pinnatisectum* (+) *tbr* (Sidorov et al. 1987), *S. commersonii* (+) *tbr* (Cardi et al. 1999), *Solanum sanctae*-*rosae* (+) *tbr* (Harding and Millam 2000) and *Solanum chacoense* (+) *tbr* potato hybrids (Chen et al. 2013). No cpDNA alterations were noticed (Pehu et al. 1989; Xu and Pehu 1993; Cardi et al. 1999) in contrast to mtDNA, where recombination is often observed (Lössl et al. 1994). In some cases, no recombination of mtDNA was detected (Przetakiewicz et al. 2007).

The goal of this study was to determine the composition of nuclear and cytoplasmic genomes of *S.* × *michoacanum* (+) *S.tuberosum* [*mch* (+) *tbr*] somatic hybrids and autofused 4*xS.* × *michoacanum* (autofused 4*xmch*) lines obtained from autofusion of 2*xmch*. DArT markers and markers specific to cpDNA and mtDNA were used for these analyses. We aimed to explain why resistance to *P. infestans* from *mch* was poorly transmitted to *mch* (+) *tbr* somatic hybrids.

## Materials and methods

### Plant materials

A total of 97 tuber-bearing interspecific somatic hybrids *mch* (+) *tbr* from five fusion combinations [*mch*/8 (+) dHBard (*a*), *mch/8* (+) cultivar (cv.) Rywal (*b*), *mch*/39 (+) cv. Rywal (*c*), *mch*/39 (+) DG 81–68 (*d*) and *mch*/39 (+) dHBard (*e*), 11 (AF1–AF10 and MS96) tuber-bearing autofused 4*xmch*] and their parental forms were used. AF1–AF10 regenerated from protoplasts of *mch*/8, and MS96 regenerated from *mch*/39 protoplasts. Hybrid plants were derived previously from a protoplast electrofusion between two diploid clones of *mch* [99–12/8 (*mch*/8) and 99–12/39 (*mch*/39)], two diploid potato clones [DG 81–68 and dHBard] and cv. Rywal as described previously by Smyda et al. (2013). Clones *mch*/8 and *mch*/39 were derived from *mch* [accession VIR5763 from the N. I. Vavilov Research Institute of Plant Industry (VIR) potato collection (Zoteyeva et al. 2012)] and both were resistant to *P. infestans*. The *mch*/8 was the parental form of mapping population used for locating the gene for resistance to *P. infestans*, *Rpi*-*mch1,* to the potato chromosome VII (Śliwka et al. 2012a). The CAPS marker C2_At1g53670 was the closest to the *Rpi*-*mch1* gene (located 5.7 cM from it), and was used as a diagnostic marker among six somatic hybrids originating from *mch*/8 parent. The gene(s) underlying the late blight resistance of the parental form *mch*/39 are not mapped and no markers are available to track this resistance in the remaining 91 hybrids. DG 81–68 and dHBard diploids from Plant Breeding and Acclimatization Institute – National Research Institute (IHAR-PIB) Młochów and Polish cv. Rywal, were susceptible to late blight. DG 81–68 was a hybrid of *tbr*, *S. chacoense* and *S. yungasense*; it was a male fertile, producing functional 2*n* male gametes. Dihaploid Bard derived from cv. Bard was male sterile; however, it functioned well as a seed parent. Cv. Rywal was resistant to PVY (Szajko et al. 2008). A total of 97 somatic hybrids were named from MS1 to MS95 and MS97 to MS98. Four hybrids from the *a* combination, 39 from *d* and 44 from *e* were tetraploid. The ploidy levels of two *b* and eight *c* hybrids were greater than 4*x*. The ploidy level was evaluated by counting chloroplasts in the guard cells. The mean number of chloroplasts in the pair of guard cells was assumed to be 11.2 (range 7.5–14.0) for diploids, 14.4 (range 10.7–19.0) for triploids and 19.7 (range 16.0–25.7) for tetraploids (Rothacker and Junges 1966). The potato genotypes used as a standard for multiplex PCR assessment of the type of cytoplasmic genome included cv. Early Rose (T type), cv. Maris Piper (A type), IVP48 (P type) and PW 363 (D type). Cultivars Newskij, Early Rose and Stobrawa were included in mtDNA analysis as a standard for α, β and γ types, respectively.

### DNA extraction and DArT analysis

Genomic DNA was extracted from 1 g of fresh, young leaves of greenhouse-grown plants using the DNeasy Plant Maxi Kit (Qiagen, Hilden, Germany). The DNA quantity was determined with a BioPhotometer plus (Eppendorf). The quality of DNA was assessed on 1.5 % agarose gels. The DArT analysis was performed in Diversity Array Pty Ltd. Canberra, Australia, as described for *mch* and *S. ruiz*-*ceballosii* by Śliwka et al. (2012a, b) based on protocols for other plant species (Jaccoud et al. 2001; Wenzl et al. 2004; Akbari et al. 2006). To maximize the output of the analysis, samples were processed by two panels: one dedicated to wild potato species and one representing clones of *tbr.* The obtained results were presented in binary scores (0/1). Markers were selected if they were polymorphic between *mch* and *tbr* parents of each fusion combination and passed the following quality control parameters: *p* value, call rate, PIC and discordance. Localization of individual markers to the appropriate chromosome was performed based on comparison with DArT maps of diploid potato species: *S. phureja* (Sharma et al. 2013), *mch* (Śliwka et al. 2012a), *S. ruiz*-*ceballosii* (Śliwka et al. 2012b) and three diploid hybrids of *tbr* (Sołtys-Kalina et al. 2015; Śliwka et al. 2016; data unpublished, personal communication with Agnieszka Hara-Skrzypiec). To determine the nuclear genome composition of *mch* (+) *tbr* somatic hybrids, preserved and deleted markers were described. Preserved markers were markers present in *mch* or *tbr* parental genome and present in somatic hybrid genome. Deleted markers were present in one of the parental forms, but absent in somatic hybrid genome.

### Late blight resistance assessment

Resistance to foliage blight of somatic hybrids and autofused 4*xmch* was assessed in laboratory tests using detached leaf tests. Somatic hybrids, autofused 4*xmch*, parental forms and standard cultivars were tested together in each test. Tests were performed on two different dates and in two replicates. In 2009 and 2010, plants tested for late blight resistance were obtained from in vitro. In 2011, 2012 and 2013, the tested plants were grown from tubers. The results of late blight resistance tests in 2009–2011 were published by Smyda et al. (2013). One to six leaflets from a single leaf were scored in a single replicate. Resistance was evaluated on a scale of 1–9, where 9 was the most resistant. In total, about 24 leaflets per genotype were scored in 2009–2013. A mean resistance score ≥6 indicated genotypes resistant to *P. infestans* (Śliwka et al. 2012a). Two isolates of *P. infestans*, i.e., MP847 and MP921, were used for spray inoculation with a concentration of 50 sporangia/µl. Both isolates originated from pathogen collection of IHAR–PIB, Młochów. The characteristics and preparation of isolates and details of the testing procedure were described by Śliwka et al. (2012a) and Smyda et al. (2013).

### PCR and restriction digestion

Cytoplasm types were examined in somatic hybrids and their parental forms using a molecular marker system elaborated by Hosaka and Sanetomo (2012). In multiplex PCR, the following markers were amplified: T, S, SAC and A chloroplast-specific markers and the D mitochondrial DNA marker (Sanetomo and Hosaka 2013). In addition to grouping the cytoplasm into six types, the evaluation was supplemented using the additional mitochondrial markers ALM_4 and ALM_5 in the PCR reaction. Thus, W type cytoplasm was classified into three mitochondrial types: W/α, W/ß and W/γ. Additionally, for evaluation of changes in mtDNA, three pairs of SCAR primers: nad1B/nad1C, ALM_1/3 and ALM_6/7 (Chimote et al. 2008), and a pair of CAPS primers: pumD (Scotti et al. 2007) specific to mtDNA were applied. Genomic DNA was used in PCR amplification with cpDNA- and mtDNA-specific primers. The multiplex PCR reaction (as described by Hosaka and Sanetomo 2012) was performed in a T3000 thermocycler (Biometra GmbH) in a total volume of 20 µl reaction mixture containing 2 µl of 10× buffer including 20 mM MgCl_2_ (Fermentas Life Sciences, Thermo Fischer Scientific, Inc.), 0.5 mM of each dNTP, 2 µM primer T, S and SAC and 3 µM primer D and A, 0.05 U/µl DreamTaq polymerase (Fermentas Life Sciences, Thermo Fischer Scientific, Inc.) and 30 of ng DNA template. The PCR parameters for multiplex PCR were 95 °C for 10 min followed by 35 cycles at 94 °C for 30 s, 60 °C for 30 s, 72 °C for 60 s and one final extension at 72 °C for 5 min. Digestion of the amplicons with restriction endonuclease *Bam*HI (Fermentas Life Sciences, Thermo Fischer Scientific, Inc.) was performed according to producers’ protocol at 37 °C for 3 h. MtDNA was divided into three groups, i.e., α, β, γ, based on the presence or absence of two DNA fragments of the ALM_4 and ALM_5 marker: 2.4-kbp or 1.6-kbp bands (Lössl et al. 2000; Chimote et al. 2008; Hosaka and Sanetomo 2012). The PCR amplification of ALM_4 and ALM_5 markers was performed in a volume of 20 µl consisting of 2 µl of 10× buffer including 20 mM MgCl_2_ (Fermentas Life Sciences, Thermo Fischer Scientific, Inc.), 3 µM of each ALM_4 and ALM_5 primers, 0.05 U/µl DreamTaq polymerase (Fermentas Life Sciences, Thermo Fischer Scientific, Inc.) and 30 ng of DNA template. A thermal profile of the PCR reaction included one cycle of 95 °C for 10 min followed by 35 cycles at 94 °C for 30 s, 57 °C for 60 s, 72 °C for 90 s and one final extension at 72 °C for 5 min.

The amplicons of multiplex PCR were separated in 1.5 % high resolution agarose gels (EURx, Ltd., Gdańsk, Poland). PCR products of ALM_4 and ALM_5 were separated in 1.5 % standard agarose gels. PCR products were stained with ethidium bromide and assessed under UV light after electrophoresis in 1× TBE buffer (Tris-Borate-EDTA). A 100-bp DNA ladder (Invitrogen) was used to determine marker sizes.

To determine whether the multiplex PCR detects different types of cytoplasmic DNA mixed in one probe, bulked probes with different configurations of cytoplasmic types: W + T; T + D; W + D and W + T + D were made. DNA of each cytoplasmic types was mixed in a ratio 1:1, and in the last configuration 1:1:1. All PCR reactions were repeated at least twice, and consistent results were recognized as reliable.

## Results

### Nuclear genome composition of somatic hybrids

**Fig. 1:**
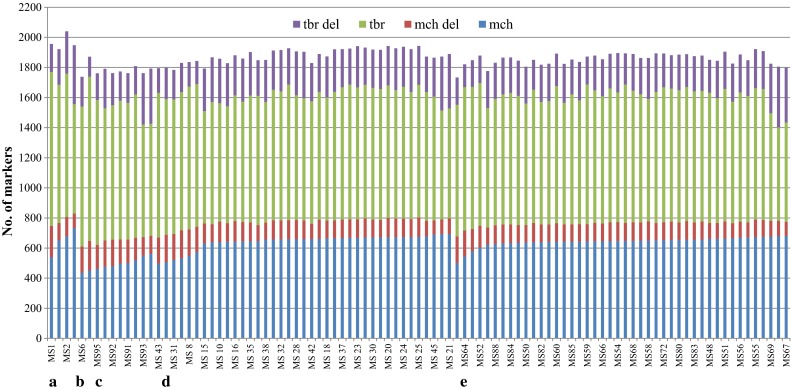
Nuclear genome composition in the number of DArT markers of 97 somatic hybrids from five fusion combinations: *a*, *b*, *c,* *d* and *e.* Results were obtained based on all polymorphic DArT markers with known and unknown chromosomal locations. Somatic hybrids are named from MS1**–**MS95 and MS97**–**MS98

The nuclear genome composition of somatic hybrids and autofused 4*xmch* was determined based on DArT markers. Data on 5358 DArT markers were obtained from DArT analysis. After quality control and selection of polymorphic markers on a parental level, an average of 2080 markers (in the range of 2011–2231 among five fusion combinations) were useful for analysis of somatic hybrid nuclear genome composition (Table 1). Primary data of all polymorphic DArT markers specific to *mch* and *tbr* parental genomes revealed that the majority of the markers were preserved in 97 somatic hybrid genomes; however, portions of both *mch* and *tbr* markers were present in parental forms, but absent in somatic hybrids genomes in every fusion combination. Deletion of markers specific to *mch* and the presence of markers specific to *tbr* in autofused 4*xmch* determined the rate of error of the applied method. The autofused 4*xmch* genome retained nearly all *mch*-specific markers. On an average, 0.65 % of the markers (2.8 out of 429.6) characteristic for *mch* were absent, whereas added markers characteristic for *tbr* genome averaged at 0.82 % (5.5 out of 665.4). All the 97 analyzed somatic hybrids from five fusion combinations contained markers from both parents, but with different dosages, and their genomes differed from each other within combinations and among combinations (Fig. 1). The percentage of deletion of *mch* markers in every fusion combinations was between 17.5 and 29.6 %. The percentage of *tbr* marker deletions ranged from 13.9 to 23.4 %. The ranges of deletion of *mch*-specific markers of individual hybrids in respective combinations were 97–208 in *a*, 174–198 in *b*, 120–159 in *c*, 95–185 in *d* and 93–174 in *e*. Conversely, the ranges of deletion of *tbr*-specific markers of individual hybrids were 186–392 in *a*, 132–197 in *b*, 177–367 in *c*, 154–361 in *d* and 152–403 in *e* (Fig. 1).

**Table 1:** The number of DArT markers useful for analysis of somatic hybrids nuclear genome composition from five *mch* (+) *tbr* fusion combinations

Fusion combinations		Ploidy level	Somatic hybrids	Number of polymorphic markers		Number of polymorphic markers with known chromosomal location	
				Total	Specific to *mch*/*tbr*	Total	Specific to *mch*/*tbr*
*a*	*mch*/8 (+) dHBard	4*x*	4	2121	852/1269	1580	566/1014
*b*	*mch/*8 (+) cv. Rywal	>4*x*	2	2024	694/1330	1568	450/1118
*c*	*mch*/39 (+) cv. Rywal	>4*x*	8	2011	701/1310	1513	434/1079
*d*	*mch*/39 (+) DG 81-68	4*x*	39	2011	810/1201	1536	533/1003
*e*	*mch*/39 (+) dHBard	4*x*	44	2231	791/1440	1505	513/992

**Fig. 2:**
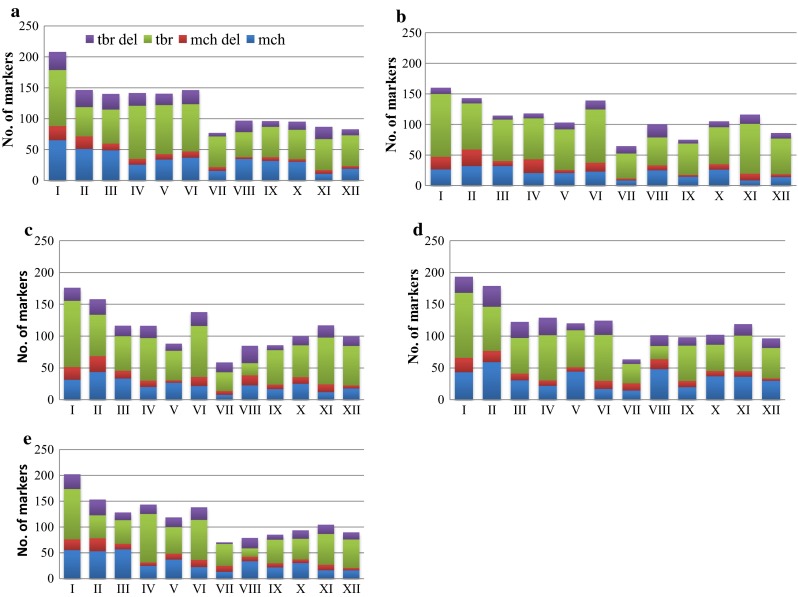
Average composition in the number of DArT markers of 12 chromosomes of somatic hybrids from fusion combinations

To analyze the chromosomal composition of somatic hybrids, polymorphic markers were compared with markers of known chromosomal location from the existing DArT maps for potato. Of five sets of polymorphic markers each greater than 2000, 1505 up to 1580 DArT markers with a known chromosomal location were identified in particular fusion combinations (Table 1). The average numbers of mapped markers within chromosomes in particular combinations were different and reached from 58 to 77 markers only for chromosome VII up to 160–208 markers for chromosome I (Fig. 2, see additional file 1). Analyzed somatic hybrids indicated differentiation in the composition of individual chromosomes within and among fusion combinations. A repeated pattern of chromosomal changes of the somatic hybrid genome was also noted. Present and absent markers from both donor genomes were spread on all chromosomes. Similarities in the composition of the chromosomes were noted among combinations at the level of present and deleted markers specific to *mch* and *tbr*, which was confirmed by analysis of rank correlation at *p* < 0.05. The *b* combination was excluded from the analysis, as it was represented by two hybrids only. The obtained correlations were significant. The strongest correlation was between mean number of *mch* deletions in particular chromosomes of combination *a*, and the mean number of *mch* deletions in particular chromosomes of combination *e* (*r* = 0.89). The remaining correlations were between *r* = 0.69–0.83 (between combinations *a* and *c*; *c* and *d*, respectively). The rank correlation of *tbr* deletions was between *r* = 0.58 (between combinations *c* and *d*) and *r* = 0.86 (between combinations *a* and *e*). The rank correlations of present markers specific to *mch* and *tbr* parental forms in particular chromosomes between combinations were *r* = 0.64–0.91 (between combinations *d* and *e*; *a* and *e*, respectively) and *r* = 0.62–0.92 (between combinations *a* and *c*; *c* and *d*, respectively), respectively. Analysis of the 12 chromosomes in fragments of 1–5 cM indicated single missing markers spread on the whole length of every single chromosome.

**Fig. 3:**
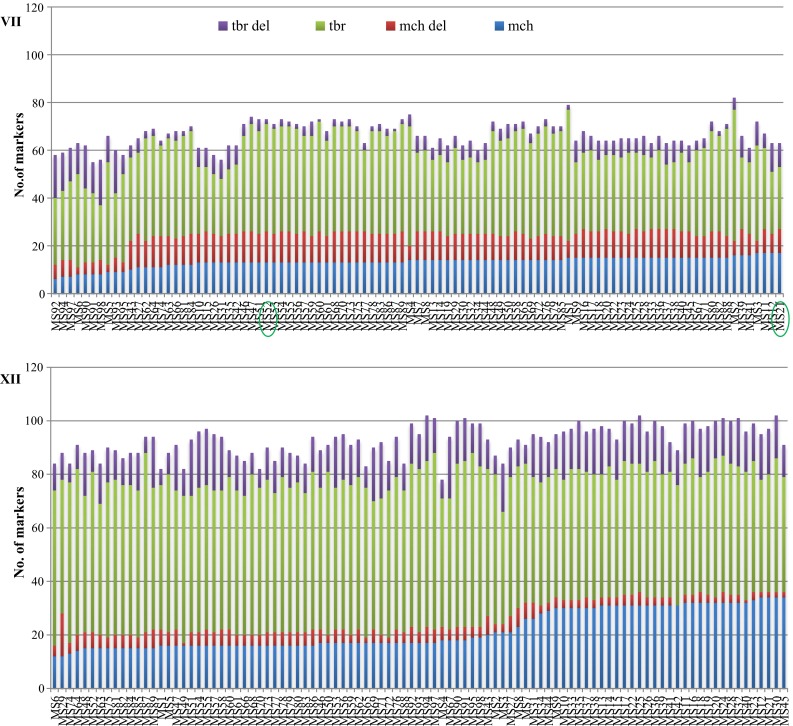
Composition in the number of DArT markers of the chromosomes VII and XII of all 97 somatic hybrids from five fusion combinations. Resistant to *P. infestans* somatic hybrids (MS52 and MS21) were marked on chromosome VII

A detailed analysis of the composition of chromosomes VII in individual somatic hybrids indicated that these chromosomes mainly contained markers specific to *tbr* parents and the majority of markers specific to *mch* were lost in most hybrids. Nearly half of the markers (37.6 %) from chromosome VII that were specific to the *mch* parent were lost in most hybrids. DArT markers located on chromosome VII originating from the map of the *mch*/8 (Śliwka et al. 2012a) were absent in present analysis. In contrast, the lowest number of *mch* deletions was observed for chromosome XII (Fig. 3).

### Resistance to *P. infestans*

**Fig. 4:**
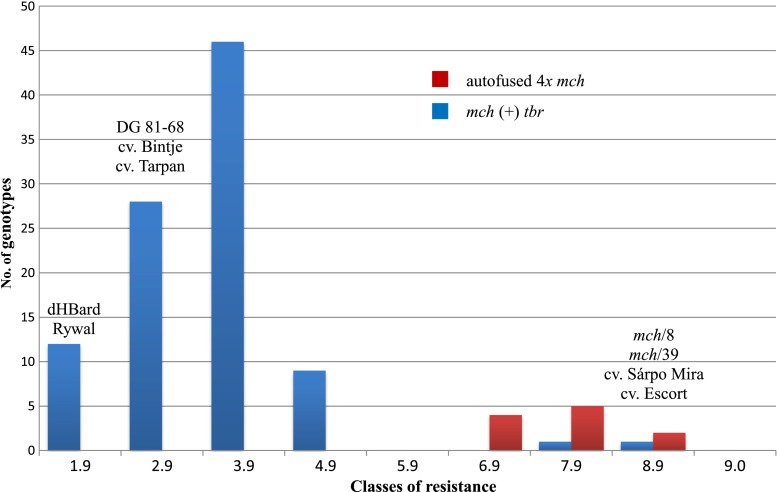
Resistance to *P. infestans* of somatic hybrids *mch* (+) *tbr* (*blue*) and autofused 4*x* *mch* (*red*) along with their parental forms and standard cultivars evaluated in a detached leaf assay (in years 2011–2013) using a scale from 1 to 9, where 9 indicated resistant

Resistance to *P. infestans* was assessed twice a year on two different dates for each somatic hybrid from 2009 to 2013. The results of testing from 2009 to 2011 were published previously (Smyda et al. 2013). The parental forms and four standard cultivars were included in every test (Fig. 4). The average resistance of *mch* parental forms were 8.4 and 8.1 for *mch*/8 and *mch*/39, respectively. DG 81–68, dHBard and cv. Rywal were susceptible to *P. infestans* with scores of 2.9, 1.3 and 1.5, respectively. The cut off separating resistant *mch* genotypes from susceptible genotypes was set at a score of 6 (Śliwka et al. 2012a) in a scale ranging from 1 to 9, where 9 is the most resistant. According to this criterion, among 97 somatic hybrids and 11 autofused 4*xmch*, two somatic hybrids (MS21 and MS52) and all autofused 4*xmch* were resistant to *P. infestans* (Fig. 4). Six susceptible to *P. infestans* somatic hybrids, originating from *mch*/8 parent (*a* and *b* combinations) did not show the presence of the C2_At1g53670 marker which was published previously (Smyda et al. 2013). All the 10 resistant autofused 4*xmch* plants derived from *mch*/8 show the presence of applied diagnostic marker. C2_At1g53670 marker was absent in *mch*/39 genome and was not diagnostic for somatic hybrids from *c*, *d* and *e* fusion combinations as well as for the autofused MS96 plant derived from *mch*/39.

### Types of somatic hybrid cytoplasmic genomes

**Fig. 5:**
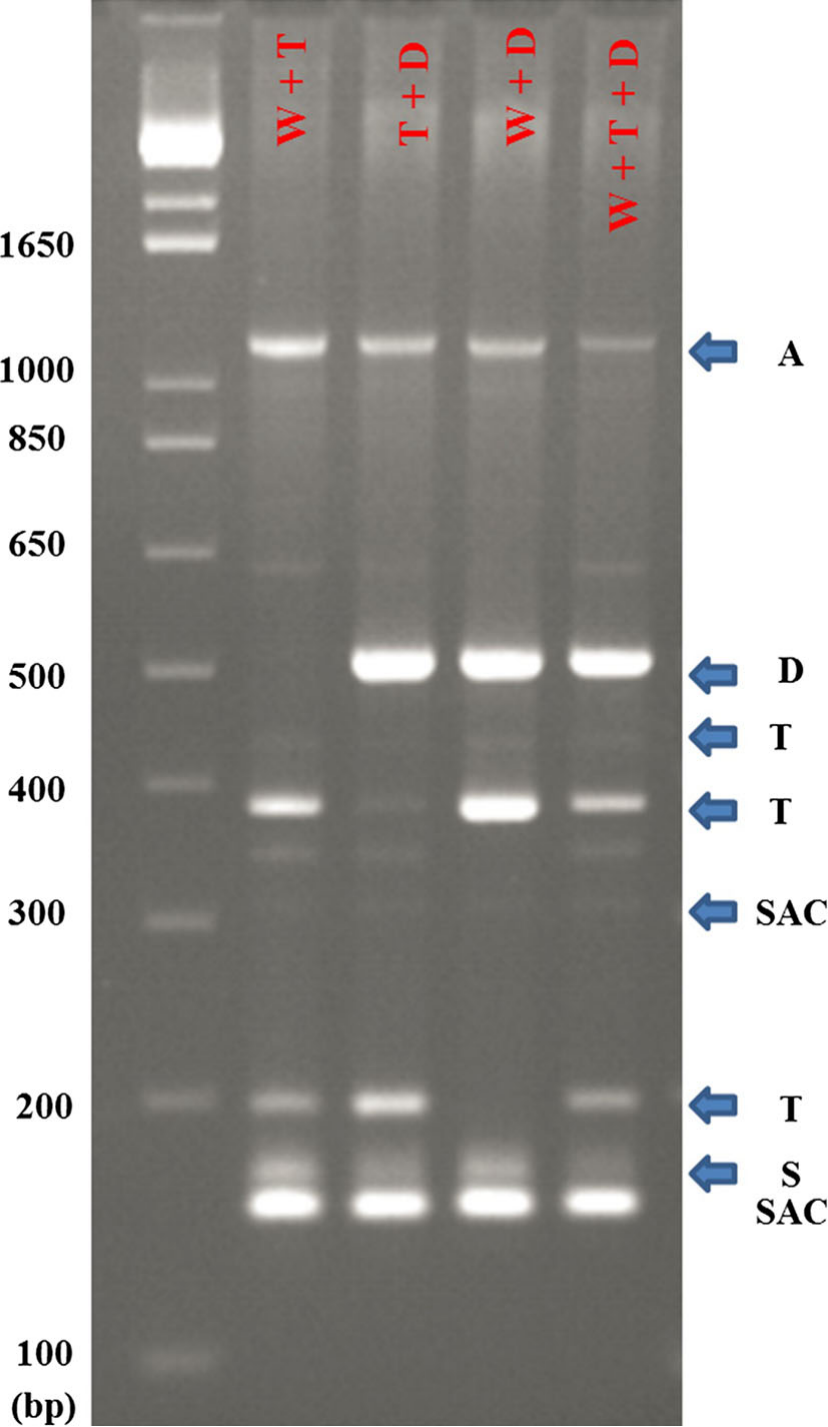
Bulked DNA probes with different configurations of cytoplasmic types amplified in one PCR reaction and digested by *Bam*HI. W + T = DNA of cv. Early Rose + *mch/*8; T + D = cv. Early Rose + PW 363; W + D = *mch*/8 + PW 363; W + T + D = cv. Early Rose + *mch*/8 + PW 363.* Arrows* indicate the markers: A, D, T, SAC and S. A 100-bp DNA ladder was used as a size marker (on the *left side*)

To determine the cytoplasmic components of the *mch* (+) *tbr* somatic hybrids, a set of five cytoplasm-specific primer pairs were used. Using the multiplex marker system (Hosaka and Sanetomo 2012), 97 somatic hybrids and their parental forms were examined. To distinguish α, β or γ mtDNA types, somatic hybrids and their parental forms were examined using the ALM_4 + ALM_5 marker (Hosaka and Sanetomo 2012). Multiplex PCR detects different types of cytoplasmic DNA mixed in one probe in various combinations (Fig. 5). The parental forms of somatic hybrids differed in the type of cytoplasmic DNA: *mch*/8 and *mch*/39 belonged to W (W/ß) type, DG 81–68 and cv. Rywal were T (T/ß) type, and dHBard was D (W/α) (Table 2). All analyzed somatic hybrids had only one type of cytoplasmic DNA that was specific to one of the parental components. The segregation of cytoplasmic types T:W in two combinations (*c* and *d*) was 1:1. Two genotypes of *b* were of W (W/ß) type. The four somatic hybrids of *a*, and the 44 somatic hybrids of *e* combinations were cytoplasmic type D (W/α) (Table 2). To reveal the potential changes in mtDNA, we applied four additional mtDNA specific markers: nad1B/nad1C, ALM_1/3, ALM_6/7 and pumD. No marker was polymorphic between fusion components, which disqualified these markers from further analysis. Comparison of cytoplasmic DNA types between in vitro plants in 2009 (directly after hybridization) and plants grown from tubers in 2013 using multiplex PCR did not indicate any differences. The hybrids with D type cytoplasm had slightly but significantly increased levels of DArT markers specific to *mch* (on average, 84.4 % of *mch* markers retained, 15.6 % of *mch* markers deleted) compared with T type (on average, 81.7 % of *mch* markers retained, 18.3 % of *mch* markers deleted) or W type (on average, 81.6 % of *mch* markers retained, 18.4 % of *mch* markers deleted), which was confirmed by the analysis of variance (*p* = 0.0058).

**Table 2:** Cytoplasmic types of the parental clones and somatic hybrids

	Type of cytoplasm			No. of individuals
	W (W/ß)	T (T/ß)	D (W/α)	
Components of somatic fusion				
*mch*/8	**+**			
*mch*/39	**+**			
DG 81-68		**+**		
dHBard			**+**	
cv. Rywal		**+**		
Fusion combinations				
*a*			4	4
*b*	2			2
*c*	4	4		8
*d*	20	19		39
*e*			44	44
Total	26	23	48	97

## Discussion

### Nuclear genome composition

This study demonstrated for the first time that DArT markers linked with available DArT genetic maps and a physical map of potato were useful method to assess the composition of 97 potato somatic hybrids. The DArT system allowed us to study the detailed composition of genomes of somatic hybrids from five fusion combinations, a composition of particular somatic hybrids and their 12 chromosomes. Moreover, it is a reliable strategy with the level of error less than 1 % (2–5.5 markers) calculated based on nuclear genome composition of autofused 4*xmch*. In our analysis, of 2000 DArT markers that were polymorphic between parents in each fusion combination and greater than 1500 markers with known chromosomal location were distributed over the whole length of all 12 chromosomes. Our results revealed that the nuclear genome composition of individual somatic hybrids was diversified with a predominance of *tbr* specific markers (1201–1440) in comparison to *mch* specific ones (694–852) (Table 1). More information obtained for *tbr* parental genome could be explained as a predominance of DNA fragments specific to *tbr* in DArT technology.

DArT markers also occurred efficient for bin mapping of tomato genomic regions of *Solanum lycopersicum* and *Solanum* pennellii in the 66 introgression lines (ILs) (van Schalkwyk et al. 2012). These markers were efficient for genome analysis and bin mapping, as 990 clones were identified and classified as polymorphic markers between parents (van Schalkwyk et al. 2012). In contrast, the nuclear genome constitution of 11 *S. acaule* (+) *tbr* somatic hybrids was characterized by nuclear RFLP markers from 49 loci (Yamada et al. 1998). Three *S. brevidens* (+) *tbr* somatic hybrids analyzed by RAPD markers provided information on approximately 99 points in the genome (Polgar et al. 1999). Chen et al. (2013) characterized the nuclear genome of 44 *S. chacoense* (+) *tbr* somatic hybrids with 108 SSR markers; 317 nuclear alleles were detected, of which 268 were polymorphic and distributed over all 12 chromosomes.

Chromosome elimination is often observed in somatic hybrids between wild and cultivated potato (Orczyk et al., 2003). The elimination of chromosomes from wild components from *S. pinnatisectum* (+) *tbr* (Menke et al. 1996), *S. phureja* (+) *tbr* (Pijacker et al. 1989) and *S. acaule* (+) *tbr* (Yamada et al. 1998) hybrids were observed. Our results indicated that specific losses of both homologous chromosomes from every pair of chromosomes did not take place, because markers specific to both parents were present in all 12 chromosomes of five combinations (Fig. 2). This is in agreement with the DNA analysis of *S. brevidens* (+) *tbr*, which indicated that specific losses of entire chromosomes of the wild parent did not occur during the plant regeneration process (Polgar et al. 1999). However, our results did not exclude the loss of one homologous chromosome and duplication of the other one from a pair. Detailed analysis of individual chromosomes in each 1–5 cM fragment indicated the deletion of single markers, distributed through whole length of every chromosome, with no visible deleted segments. Another explanation could be that in somatic hybrid genomes the methylation pattern has been reset as a result of hybridization procedure, and in regenerating hybrids due to the presence of both genomes the parental methylation patterns were not reconstructed. DArT technology uses a *Pst*I enzyme, which is methylation-sensitive, and therefore, could have digested the hybrid DNA differently, according to the new methylation pattern. More experiments are needed to confirm that hypothesis.

In our study, the composition of chromosomes within each fusion combination was diverse; however, the composition of particular chromosomes among four fusion combinations was similar. This finding was confirmed by a significant (at *p* < 0.05) statistical rank correlation for the number of present and deleted markers in respective chromosomes. In each combination, the lowest number of markers was always noticed on chromosome VII (58–77), and the highest number was noted on chromosome I (160–208). The remaining ten chromosomes in every fusion combination consisted of an increased number of markers (82–178) compared with chromosome VII, but contained fewer markers than chromosome I. The concentration of markers located on each chromosome was the derivative of the density of previous DArT maps. The *S. phureja* diploid physical map (Sharma et al. 2013) consists of 2530 markers, including 1827 DArT markers. The DArT map for *mch* (Śliwka et al. 2012a) includes 846 DArT markers and the map for *S.ruiz*-*ceballosii* contained 1603 DArT markers (Śliwka et al. 2012b). Next three maps were constructed in IHAR–PIB, Młochów and consisted of 1597, 1420, and 1370 DArT markers, respectively (Sołtys-Kalina et al. 2015; Śliwka et al. 2016; data unpublished, personal communication with Agnieszka Hara-Skrzypiec). The chromosomes richest in markers were chromosomes I (133–230 markers) and II (75–260 markers) in all six analyzed DArT maps. The smallest number of markers was observed on chromosome IV in two maps (Sołtys-Kalina et al. 2015; data unpublished, personal communication with Agnieszka Hara-Skrzypiec) (29 and 48 markers), and the map for *S.ruiz*-*ceballosii* (74), on chromosome V (86) in the third map (Śliwka et al. 2016), on chromosome VII (28) in the *mch* map, and on chromosome XII (36) in the physical map (Sharma et al. 2013). The results of analysis of *mch* (+) *tbr* somatic hybrid genomes corresponded well with these data. Obtained differences in marker numbers per chromosome may be caused by the greater polymorphism of some chromosomes (e.g., chromosome I), their physical size, or the fact that more information was available from previous maps. The karyotype of potato arranged on the basis of chromosome lengths indicated that chromosome I was the longest, and the shortest was chromosome XII (Dong et al. 2000). In the potato physical map, chromosome I was also the longest (88.7 Mb), and chromosome XI was the shortest (45.5 Mb) (Sharma et al. 2013). An additional explanation of differences in the number of markers observed among chromosomes involves the composition of genome representation used in DArT technology. It is possible that DNA fragments specific to chromosome I predominated the remainder of the markers.

### Transmission of resistance to late blight

Only two hybrids from 97 tested were resistant to late blight. There is a question why the remaining 95 hybrids did not exhibit resistance of the *mch* fusion component. The *Rpi*-*mch1* gene is located on distal part of chromosome VII of *mch*/8 parent (Śliwka et al. 2012a). It could have been inherited by the six hybrids (combinations *a* and *b*) originating from this parent, but the closely linked marker C2_At1g53670 (5.7 cM) was absent in them and these hybrids were susceptible, which supports the assumption of the deletion of one homologous chromosome, namely, chromosome VII. The markers linked to the *Rpi*-*mch1* gene were useless as diagnostic markers among resistant somatic hybrids originating from *mch*/39 (MS21 and MS52), because the gene(s) underlying the late blight resistance of this parental form remain unidentified. However, *mch*/8 and *mch*/39 parental forms are related, originated from the same accession number and because of that the structure of chromosome VII of all somatic hybrids was analyzed. In our study, this chromosome was poor in the DArT markers, markers from the *mch* genetic map (Śliwka et al. 2012a) were absent and the chromosome VII composition was strongly dominated by *tbr* (23–55) markers compared with *mch* (6–17) markers. The average number of alleles specific to a *mch* parent from chromosome VII was from 1 to 5 per analyzed fragment of 5 cM. The resistant to late blight somatic hybrid MS21 contained the highest number of DArT markers specific to the *mch* genome. The number of markers specific to *mch* and *tbr* and their deletions in the second resistant hybrid MS52 was similar to that of other hybrids susceptible to late blight. There were deletions of single markers along the whole length of chromosome VII (from 0 to 88.46 cM) with no deletions of chromosomal segments, which also could be explained by the reset of somatic hybrids’ methylation pattern (described above). This will be the subject of further research. However, the high input of deleted *mch* DArT markers (average 37.6 %) as well as, in case of *mch*/8-derived hybrids, the absence of the CAPS marker may indicate the loss of the entire homologous chromosome with the locus *Rpi*-*mch1* given that the locus *Rpi*-*mch1* was present in the parental form *mch/*8 in a heterozygous condition. Lack of resistance could also result from the loss of genetic factors other than the gene *Rpi*-*mch1*. In susceptible forms predominantly noted in our studies, the expression of a resistance gene might have been silenced. The lack of resistant somatic hybrids or the low frequency of the expected level of resistance to late blight in the somatic hybrid genome were previously noted (Thieme et al. 1997; Rasmussen et al. 1998; Bidani et al. 2007; Szczerbakowa et al. 2010; Polzerová et al. 2011). Such phenomena could be explained by a ‘dilution effect of non-resistance genes’, which means that expression of a resistant gene from one parental form in somatic hybrids genome is reduced by the presence of non-resistance genes from the other component of the somatic fusion or as a different genome-dosage; chromosomal instability; preferential elimination of some chromosomes; somaclonal variation in an early stage of regeneration, which generated gene mutations in the nuclear and cytoplasmic DNA; translocations and deletions (Thieme et al. 1997; Rasmussen et al. 1998). The majority of these phenomena will most likely never be overcome given the very random character of this process (Orczyk et al. 2003).

### Cytoplasmic diversity of somatic hybrids

We assessed the variability of cpDNA and mtDNA in 97 studied somatic hybrids with six markers that were polymorphic on a parental level. Four markers were specific to cpDNA, and two were specific to mtDNA (Hosaka and Sanetomo 2012). Sorting of cpDNA and mtDNA in various potato somatic hybrids indicated random or preferential patterns according to the concept of alloplasmic compatibility (Orczyk et al. 2003). In the present study, no hybrids with mixed cpDNA and mtDNA were noted, and we observed both random and non-random types of sorting of organellar DNA. Non-random segregation was in two combinations, i.e., *a* and *e*, where predominance of the D type cytoplasmic DNA was observed. Statistical analysis indicated a significant positive correlation between cytoplasmic DNA type D and the percentage of nuclear DArT markers specific to the *mch* parent. These data suggested that D type cpDNA and mtDNA are perhaps more compatible with nuclei containing more *mch*. Plastid and mitochondrial proteins are encoded by nuclear genes, and the disruption of organellar interactions may result in nucleo-cytoplasmic incompatibility and cause reduction of survivability of somatic hybrids (Leon et al. 1998; Orczyk et al. 2003). Somatic hybrids from *c* and *d* combinations contained organelles from one or a second parent at random in types W or T. In *S. brevidens* (+) *tbr* (Xu and Pehu 1993) and *S. sanctae*-*rosae* (+) *tbr* (Harding and Millam 2000), *S. chacoense* (+) *tbr* (Chen et al. 2013) somatic hybrids, the chloroplast genome was inherited from one of the parents. There is a higher frequency of mtDNA recombination than cpDNA in the somatic hybrids, (Chen et al. 2013). Chen et al. (2013) observed a range of mitochondrial rearrangements caused by the structure of mitochondria with a large number of repeated sequences. In our study, we did not recognize both types: α and β of parental mtDNA in genomes of somatic hybrids from combinations *a* and *e*. There were no differences between mtDNA in the remaining combinations (both parental forms were β). We cannot exclude recombination in mitochondrial structure based on two mtDNA specific markers. In our study, based on applied system the cpDNA was without rearrangements in its structure. Our analysis of DNA from 2009 to 2013 indicated that there were no differences between propagation stages, suggesting stability of the cytoplasmic genome of somatic hybrids and indicating the elimination of cytoplasmic DNA occurred at an early stage of regeneration.

#### **Author contribution statement**

PSD: participated in the design of the study, performed the resistance testing, prepared DNA for DArT analysis, took part in the analysis of DArT results, molecular analysis of organellar DNA and drafted the manuscript; JŚ: participated in the design of the study and its coordination, in writing the manuscript and analysis of DArT results; IWF: performed cytological analysis; HJ: participated in the resistance testing and in writing the manuscript; EZG: participated in the design of the study and its coordination, and took part in writing.

## Electronic supplementary material

Below is the link to the electronic supplementary material.

Supplementary material 1 **Additional file 1:** Composition in the number of present and absent DArT markers, specific to *mch* and *tbr* parent of the particular 12 chromosomes of 97 somatic hybrids from five fusion combinations: *a* (N = 4; MS1-MS4), *b* (N = 2; MS5-MS6), *c* (N = 8; MS90-MS95 and MS97-MS98), *d* (N = 39; MS7-MS45), *e* (N = 44; MS46-MS89). (XLS 480 kb)

## Abbreviations

DArT: Diversity array technology
*mch* (+) *tbr*: *Solanum* **×** *michoacanum* (+) *S. tuberosum*
4*x**mch*: 4*x**S.* × *michoacanum*
*P. infestans*: *Phytophthora infestans*
cpDNA: Chloroplast DNA
mtDNA: Mitochondrial DNA
